# A Novel Patient-Derived Xenograft Model of Inflammatory Breast Cancer

**DOI:** 10.21203/rs.3.rs-9372889/v1

**Published:** 2026-05-18

**Authors:** Princess Ekpo, Monica Khattak, Tiffany Cheung, Yun Yun Su, Pushpinder K. Bains, Ivan Juric, Vladimir Makarov, Daniel Campo, Patrick McIntire, Alexander Ring, Gregor Krings, Karin List, James Hicks, Michael F. Press, Ruth Keri, Michael Stanczyk, Gigi Sengupta, Julie E. Lang

**Affiliations:** Cleveland Clinic Lerner Research Institute, Cleveland Clinic Lerner School of Medicine; Cleveland Clinic Department of Graduate Medical Education; Cleveland Clinic Department of Graduate Medical Education; University of Southern California Norris Cancer Center; University of Southern California Norris Cancer Center; Cleveland Clinic Lerner Research Institute; Cleveland Clinic Lerner Research Institute; University of Southern California Norris Cancer Center; Cleveland Clinic; University Hospital Zurich; Cleveland Clinic; Wayne State University; University of Southern California Michelson Center for Convergent Bioscience; University of Southern California Norris Cancer Center; Cleveland Clinic Lerner Research Institute, Cleveland Clinic Lerner School of Medicine; Cleveland Clinic Lerner Research Institute, Cleveland Clinic Lerner School of Medicine; Cleveland Clinic Lerner Research Institute, Cleveland Clinic Lerner School of Medicine; Cleveland Clinic Lerner Research Institute, Cleveland Clinic Lerner School of Medicine

**Keywords:** inflammatory breast cancer, IBC, patient-derived xenograft, mouse model, HER2 positive

## Abstract

**Background:**

Few models exist for studying experimental therapeutics in inflammatory breast cancer (IBC). Our study objective was to characterize a novel patient-derived xenograft (PDX) from a HER2 positive IBC patient refractory to neoadjuvant chemotherapy.

**Methods:**

We derived a novel PDX from a patient with hormone receptor negative, HER2-positive IBC refractory to neoadjuvant chemotherapy with Docetaxel, Carboplatin, Trastuzumab, and Pertuzumab (TCHP). Tumor was implanted into NOD/SCID/γ mice (NSG) and used for serial propagation of PDX. We performed short-tandem repeat (STR) profiling, plotted tumor growth curves for mice treated with alpelisib/everolimus vs. untreated, and immunohistochemistry (IHC), and performed clinical genomic assays. Paired Student’s t-tests were used to compare tumor growth curves. We used 10X Genomics for single cell transcriptome analysis of 1000 cells derived from the PDX.

**Results:**

ctDNA sequencing revealed amplifications in MYC, ERBB2 (HER2), androgen receptor (AR), PIK3CA, vMYB and loss of CDKN2A. Tumor sequencing found a H1047R mutation in PIK3CA. STR profiling showed the propagated PDX tumor matched the original tumor. The engraftment rate was 12/15 (80%) and median tumor volume doubling was 24.5 days (range 9.2–175 days) for n = 15 untreated controls. Alpelisib plus everolimus decreased tumor growth in our PDX (p = 0.006). TCHP-resistant tumor cells downregulated HER2 expression, which was re-expressed after treatment with alpelisib and everolimus.

**Conclusion:**

We established a PDX of a HER2-positive IBC tumor with a PIK3CA hotspot mutation (H1047R) refractory to TCHP. Targeting the PI3K/mTOR pathway may be useful to overcome resistance in HER2-positive IBC with a H1047R mutation in PIK3CA.

## Introduction

Inflammatory breast cancer (IBC) is one of the most aggressive forms of breast cancer, yet few models exist for studying experimental therapeutics. Non-metastatic IBC is staged as Stage III given its tendency to recur despite tri-modality therapy with upfront chemotherapy, modified radical mastectomy surgery, and external beam radiation therapy. IBC comprises 0.5 to 2.5% of invasive breast cancers diagnosed in the United States, with an incidence of 2.76 cases per 100,000 people.^1,2^ Given the rarity of IBC (less than 5% of all breast cancers), treatment decision making is based on clinical trial data that are not specific to IBC.^3^ The mean 5-year overall survival (OS) is 66% for lymph node negative and 49% for lymph node positive IBC.^4^

The MARY-X IBC mouse model, established in 1999, was the first known IBC transplantable mouse xenograft model to attempt to characterize the phenotype of IBC^5^. Unfortunately, this model is not widely available to the scientific community, and since it was derived from a triple-negative IBC patient (lacking estrogen receptor, progesterone receptor, and HER2 protein expression), it does not model the biology of HER2-positive IBC. IBC is HER2 positive 40–60% of the time, considerably higher than the overall breast cancer population.

Cell lines and animal models are the foundation of preclinical cancer research. These models provide evidence of efficacy of new therapies and shed light on the molecular mechanisms of tumor drug resistance or sensitivity. Unfortunately, since IBC is a rare, understudied disease, there exist few models in which to study new therapies.^6–10^ Such models allow scientists to dissect the molecular vulnerabilities of a tumor and have led to fundamental understanding of what makes different subtypes of breast cancer behave differently. Notably, IBC exhibits distinct clinical behavior from most cancers by spreading rapidly through lymphatic channels^11^ and causing redness of the skin^12^. Many cell lines used for IBC research have accumulated genetic drift and do not accurately represent the heterogeneity or molecular landscape of primary IBC tumors.^13–15^ These features underscore the biological distinctiveness of IBC and the necessity for subtype-specific experimental systems.

IBC tends to progress and recur at a much higher rate than non-IBC. IBC is more difficult than non-IBC to establish models since a) it is rare; b) it does not always present with a mass and clinicians have variable experience identifying the defining characteristics^12^; c) chemotherapy treatment is given prior to surgery for this disease; d) there has to be sufficient viable tumor available with scientific resources to grow tumor standing by to either implant tumor into mice or growth tumor in plastic dishes (cell or organoid culture).

The objective of our study was to establish and characterize a novel patient-derived xenograft (PDX) from a HER2 positive IBC patient refractory to neoadjuvant chemotherapy to better understand the biology of IBC resistance.

## Materials and Methods

### Inflammatory Breast Cancer (IBC) Model

IBC patients were consented under the University of Southern California (USC) IRB HS-18–00628. Tumor samples collected sterilely during surgery at Los Angeles County + USC Medical Center were stored in DMEM medium (ThermoFisher Scientific, Inc. Waltham, MA, USA) supplemented with Gibco 1x antibiotic-antimycotic solution (ThermoFisher Scientific, Inc. Waltham, MA, USA) on ice before implantation into mice. All xenograft implantations were completed within 4 hours after surgery. A total of three patients were enrolled in this study. The first two patients did not yield viable PDX. The third patient (USCIBC3) was a 52-year-old female with Stage III IBC that was ER negative (0%), PR negative (0%) and HER2 positive (FISH amplified with a HER2:CEP17 ratio of 6.88) that was refractory to neoadjuvant chemotherapy with Docetaxel, Carboplatin, Trastuzumab, and Pertuzumab (TCHP) with a tumor that was ypT3N0 on surgical pathology from modified radical mastectomy. Fresh tumor was harvested sterilely directly from the operating room and immediately placed in Dulbecco's Modified Eagle Medium (DMEM). Fresh tumor arrived at the vivarium within 30 minutes of resection. The patient’s clinical tumor sample was sent for molecular profiling by Caris Life Sciences (Phoenix, AZ). Blood from USCIBC3 patient (after neoadjuvant chemotherapy) was sent for circulating tumor DNA (ctDNA) profiling with genomic sequencing performed via collaboration with the laboratory of Dr. James Hicks, Ph.D and Fulgent (Los Angeles, CA).

### Patient Derived-Xenograft (PDX) Mouse Experiments

Six- to eight-week-old female NOD/SCID/γ (NOD.Cg-Prkdcscid Il2rgtm1Wjl/SzJ) (NSG) mice were purchased from the Jackson Laboratory (Bar Harbor, Maine, USA) and housed at the USC Zilkha Neurogenetic Institute (ZNI) vivarium. Mouse experiments were approved and performed under USC IACUC protocol 20937 with daily inspection of the mice. Tumor tissue was cut to 3×3mm pieces under sterile conditions and coated with Matrigel (Corning, New York, NY, USA). Each patient’s tumor was implanted into 5 mice. Tumor pieces were bilaterally implanted orthotopically into mice in the fourth mammary fat pads under 2 to 3% of isoflurane via nasal cone following 0.5–1mg/kg of buprenorphine subcutaneously injection. Mice were kept warm using a warming pad. For estrogen receptor positive tumors, mice were subcutaneously implanted 50ug/mL of 17β-estradiol (Sigma, St Louis, MO, USA) through silastic capsules in the necks^16^. The tumor size was measured by calipers and the growth of tumor was monitored. When the tumor volume reached 2000mm^3^ or humane endpoints were reached, the mice were euthanized by carbon dioxide-regulated chamber and cervical dislocation. The tumor was cut into 3×3mm pieces of tissue and immediately transplanted to another 3 to 5 NSG mice for propagation by survival surgery into the next generation of mice. The remaining tumor pieces were stored in 95% fetal bovine serum (FBS) supplemented with 5% DMSO in liquid nitrogen for cryopreservation. Paired Student’s t-tests were used to compare tumor growth curves. Median tumor volume doubling rate was calculated using the Schwartz formula for tumor volume doubling time (TVDT) = log2 (T2-T1)/log(V2/V1) for n = 15 untreated control mice. Mice were weighed and tumors measured using calipers weekly.

### PDX Treatment with Alpelisib and Everolimus

USCIBC3 PDX third-generation mice were used to test the response to treatment with alpelisib and everolimus versus control (n = 5 mice treated versus n = 8 mice untreated). PDX tumors were allowed to grow for 65 days until tumors reached a tumor volume of 200mm^3^. Each mouse was given 100ul of 20mg/kg of alpelisib and 10mg/kg of everolimus dissolved in 5%DMSO, 40%PEG300, 5% Tween-80 and 50% sterile distilled water daily by oral gavage with a stainless-steel bulb-tipped gavage needle for 21 days. Mice were monitored for 10 minutes post-gavage for any complications. Paired Student’s t-tests were used to compare tumor growth curves.

### Immunohistochemistry

Immunohistochemistry (IHC) of formalin-fixed, paraffin-embedded PDX tissues was performed at the University of Southern California Clinical IHC and Research laboratory of the Department of Pathology, Los Angeles, CA. Briefly, antigen retrieval was performed in citrate buffer (pH 6) in a 100°C steam bath for 20–45 minutes or EDTA buffer (pH8) for 30 minutes following deparaffinization. Primary antibodies of CD44 (156–3C11) 1:100 and Her2/neu (IHC042) 1:100 (Biocare Medical, Pacheco, CA USA); E-Cadherin (36B5) 1:25 and progesterone receptor (PR) (16) 1:100 (Leica Biosystems Inc. Grove, IL, USA); estrogen receptor (ER) (SP1) 1:200 (Abcam, Cambridge, MA, USA), EGFR (M7239) 1:50 (DAKO, Glostrup, Denmark); ALDH16A1 1:200 and LYVE1 1:5000 (Atlas Antibodies, Stockholm, Sweden); and JAK2 (Ab570) 1:50 and PIK3CA (MCM) 1:1000 (MilliporeSigma, St. Louis, MO USA); CD24 1:500 (Novus, Saint Charles, MO, USA); Phospho-Stat3 (Tyr705)(D3A7) 1:100 (Cell Signaling Technology, Danvers, MA, USA) were used to detect protein expressions on the tumor sections. All primary antibodies were incubated for 60 minutes at 37°C. Secondary antibody against rabbit HRP polymer (Biocare Medical, Pacheco, CA USA) was incubated for 30 minutes at 37°C. Chromogen DAB and Hematoxylin (Leica Biosystems Inc. Grove, IL, USA) were incubated for 10 minutes respectively for conjugation and counterstaining. IHC was performed on PDX treated with Alpelisib and Everolimus versus untreated PDX tumor tissues. Digital samples downloaded from the USC Department of Pathology Philips Digital Pathology Solutions platform and were reviewed by a board-certified pathologist and H-scores were used to assess protein expression by IHC.

### Cell Culture

Primary cancer cell isolation was performed by using Primary Cancer Culture System kit (PromoCell, GmbH, Heidelberg, Germany) according to the manufacturer’s instructions. Briefly, USCIBC3 patient’s tumor sample was minced into small pieces of approximately 1mm by scalpel at the sterile dishes. The tumor tissue was digested with Accumax solution (Sigma, St Louis, MO, USA) at a concentration of 20 mL per gram of tumor tissue for 40 minutes at the room temperature with gentle mixing. Single-cell suspensions were obtained serial through 70um and 40um filter (BD Biosciences, San Jose, CA, USA).

The cells were washed twice with primary cancer cell medium D-ACF and pelleted at 300xg centrifuge for 5 minutes at room temperature.

### Short Tandem Repeat (STR) Profiling

STR profiling of primary human tumor and PDX tumor were performed at the University of Arizona Genetics Core, Tucson, AZ.

According to manufacturer’s instructions, genomic DNA was isolated using DNeasy Blood & Tissue Kit (Qiagen, LLC, Germantown, MD, USA), and was diluted to 5ng/μL in PCR grade water. STR profiling was identified by using the PowerPlex^®^ 16 System kit (Promega, Madison, WI, USA) with 15 autosomal loci. Percent match was calculated as the Match Algorith X 100. The match Algorithm was calculated as shown below.


$$\text{Match Algorithm}=\frac{\text{SHARED ALLELES}\times 2}{\text{TOTAL ALLELES in the The Sample}+\text{TOTAL ALLELES in the Reference Sample}}$$

A match was defined by the International Cell Line Authentication Committee as a result greater than or equal to 80%.

#### 10x Genomics Single Cell Sequencing

The cell suspensions of primary human tumor and PDX tumor were prepared by digesting them in Accumax solution (Sigma, St Louis, MO, USA) at a concentration of 20 mL per gram of tumor tissue for 40 minutes at room temperature with gentle mixing and then passing through 70um and 40um filters (BD Biosciences, San Jose, CA, USA) respectively. The cell suspension was washed twice with culture medium and stored in 90% FBS with 10%DMSO in liquid nitrogen.

The frozen cell suspension was thawed in 30 mL of growth medium, centrifuged at 300 g for 5 minutes, and then resuspended in 1 mL of 1 x PBS with 0.04% BSA at the concentration of 1 × 10^6^ cells / mL. 1000 cells were sequenced per specimen.

Single-cell sequencing was performed at the University of Southern California Molecular Genomics Core using the 10x Genomics 3' v3 protocol (10x Genomics, Pleasanton, CA) on the 10x Genomics Chromium X platform.

### Single-cell RNA-seq Data Analysis

FASTQ files were uploaded to the BioTuring Ecosystem for analysis of the Seurat Object using the BBrowser X package for cell-type prediction via metareference to perform cell type annotation, differential gene expression analysis, and creation of TSNE plots. We mapped FASTQ files to the GRCh38 reference human genome using CellRanger (v7.2.0).^17^ Following mapping, we removed cells with fewer than 600 genes and/or more than 30% mitochondrial and ribosomal genes, upon which we created Seurat object using Seurat (v4.3.0).^18^ UMAPs, feature plots, dotplots, and heatmaps were made using scCustomize library.

To compare data with previously published IBC data, we used Harmony R package to integrate data.^19^ We accessed the single-cell RNA-seq datasets for 3 IBC cell lines associated with the study “JAK-STAT Signaling in Inflammatory Breast Cancer Enables Chemotherapy-Resistant Cell States” (Cancer Research, Vol. 83, No. 2) from the Gene Expression Omnibus (GEO) database on October 7, 2025.^10^ The dataset (accession number GSE163397) contains single-cell transcriptomic profiles derived from inflammatory breast cancer samples and was generated using the Illumina NextSeq 500 platform (GPL18573). We used the Wilcoxon test as implemented in Seurat’s FindMarkers() function to identify differentially expressed (DE) genes between our dataset and the 3 IBC cell lines. The output of DE analysis, sorted by log2 fold change, was used as input for gene set enrichment analysis which was performed using the GSEA function from clusterProfiler (v4.4.4) with Hallmark and Reactome gene sets.^20^ We visualized GSEA data using custom R scripts that relied on ComplexHeatmap library. We used CellChat to interrogate signaling patterns between different cell types.^21^

### ctDNA Profiling

Plasma was thawed and cfDNA was extracted with the QIAamp Circulating Nucleic Acid Kit (QIAGEN) according to the manufacturer's instructions. DNA concentration was measured using Qubit Fluorometric Quantitation (Thermo Scientific). Illumina DNA sequencing libraries were constructed with the NEBNext Ultra II DNA Library Prep Kit (New England Biolabs) according to manufacturer's instructions and barcoded with Multiplex Oligos for Illumina (New England Biolabs). The sample size distribution of both the extracted DNA and the sequencing library was measured with the Agilent 2100 Bioanalyzer (High-sensitivity DNA Assay and Kit, Agilent Technologies). The sample was sequenced at a coverage of approximately 0.3x to generate 1.2 × 10^6^ mapped reads.

Bioinformatic analysis for copy-number profiling was performed initially as previously published.^22^ Illumina sequence reads were deconvoluted based on sample barcodes and PCR duplicates were removed. The binned ratios were normalized according to guanine-cytosine (GC) content of each bin and mapped to 20,000 bins averaging 125 kbp of uniquely mapping sequence across the human genome (hg19, Genome Reference Consortium GRCh37, UCSC Genome Browser database). Read count data was segmented using the CBS segmentation algorithm and copy-number profiles were generated from segmented bin count data and presented as ratios to the genome-wide median.^23^ ctDNA fraction and ploidy were computed using the iChor package.^24^ Approximate copy number of each locus was computed from the observed ratio: O.R. × 1.83 × (1/0.38).

### Materials Transfer

De-identified frozen human tissues, human cells, PDX, and sequencing data FASTQ files were transferred from the non-profit organizations the University of Southern California to the Cleveland Clinic via Material Transfer Agreement 4275118. The source of the PDX and ownership of the PDX remains the University of Southern California, however the investigators retain the rights to publish this work. Distribution and licensing of this model requires permission from the University of Southern California.

## Results

### Clinical and genomic characteristics

**Figure 1:**
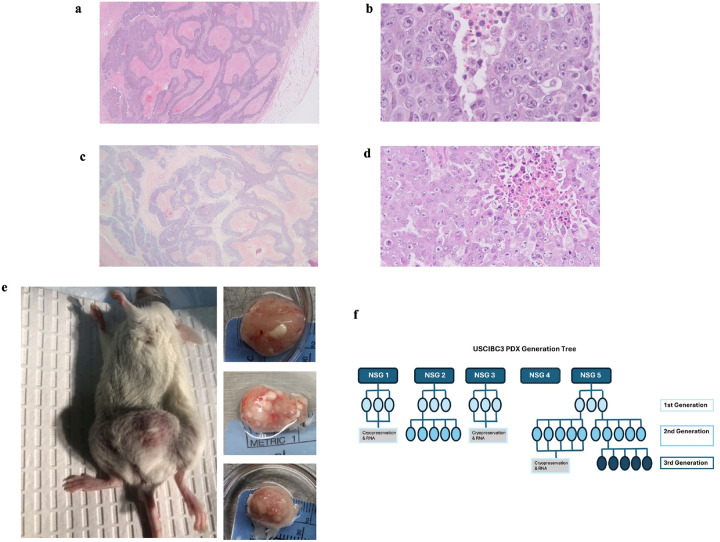
Histopathologic and Model Characterization of the USCIBC3 Patient-Derived Xenograft (PDX) Established from an Inflammatory Breast Cancer Tumor (a) Low-magnification H&E image of the patient’s primary inflammatory breast cancer (IBC) tumor showing extensive areas of necrosis. (b) High-power H&E of the same primary tumor highlighting tumor cell morphology. (c) Low-power H&E of the USCIBC3 PDX tumor demonstrating preservation of necrotic regions similar to the primary tumor. (d) High-power H&E of the USCIBC3 PDX revealing viable tumor nests and necrosis. (e) In vivo imaging of the murine model showing erythematous changes overlying the engrafted tumor, consistent with IBC features. (f) Generation schematic illustrating propagation of the USCIBC3 PDX through serial transplantation, with third-generation mice selected for molecular and histopathologic characterization. Tumor samples were implanted into NOD/SCID/γ (NSG) mice and propagated into next generation mice when tumor reached 2000 mm3.

**Figure 2:**
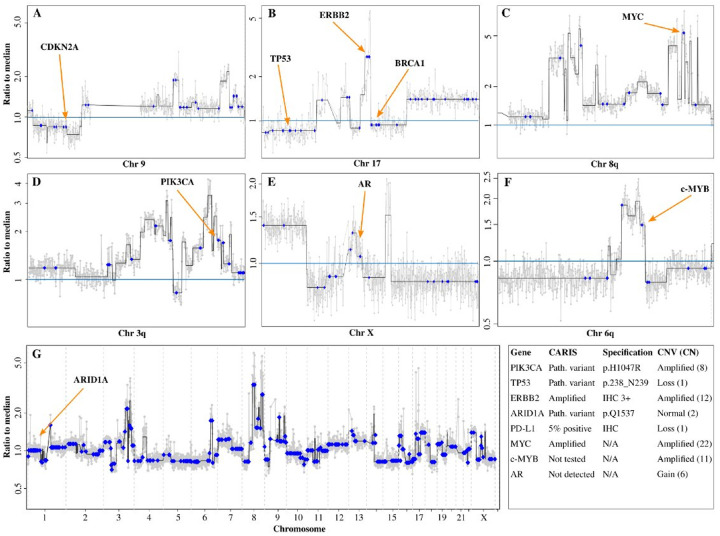
Copy Number Variation, Mutational Profile, and Tumor Fraction Analysis of ctDNA from USCIBC3 ctDNA profiling of the patient’s peripheral blood. USCIBC3 plasma had 20% tumor DNA. Copy number profiles of cfDNA from IBC: (a-f) selected chromosome segments containing functional genes as labeled (g) whole genome profile including X and Y. Gray symbols define CNV profiles plotted as ratio to median on log10 scale (Y-axis) and absolute genome position (X-axis) using 2×106 unique 50 bp reads mapped to ~5000 bins (G) or 20,000 bins (A-F). Blue symbols define a segmented data profile. Table showing results from Caris, along with imputed copy numbers of functional genes computed from the estimates of ploidy (1.83) and cfDNA fraction (0.38) derived from iChor analysis (methods).

**Figure 3:**
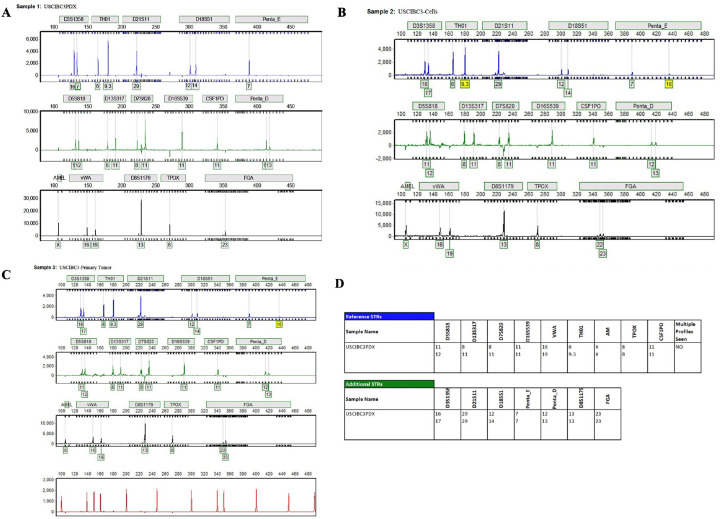
Molecular Characterization of the USCIBC3 Patient-Derived Xenograft (PDX) Short tandem repeat (STR) profiling confirmed that the USCIBC3 PDX is genetically identical to the patient’s primary HER2-positive inflammatory breast cancer, validating its origin and authenticity.

Pathology from a modified radical mastectomy (after neoadjuvant therapy) showed a high grade invasive ductal carcinoma with a diameter of 8.4 cm involving the dermis and 0/22 lymph nodes (Fig. 1A and 1B). USCIBC3 tumor induced overlying skin erythema in third-generation NSG mice (Fig. 1C–F). H&E analysis revealed that both tumors exhibited similar patterns of necrosis and comparable stromal-to-tumor ratios. Caris molecular profiling of patient tumor found the PICK3CA mutation and ERBB2 (HER2) amplification by CISH, IHC3+, PD-L1 positivity (5%) by IHC, a PIK3CA pathogenic variant detected in exon 21 at p.H1047R, MYC amplification, a TP53 likely pathogenic variant at exon 7 at p.238_N239, and a pathogenic variant in ARID1A at exon 18 at p.Q1537. AR was negative by IHC by Caris profiling (Fig. 2). The plasma of the patient from which USCIBC3 was derived had 20% circulating tumor DNA. ctDNA sequencing revealed amplifications in MYC, ERBB2 (HER2), androgen receptor (AR), PIK3CA, c-MYB and loss of CDKN2A. Consistent with the Caris findings, copy number variation (CNV) analysis of cfDNA (Fig. 2) demonstrated focal amplifications on chromosomes 8q (MYC), 17q (ERBB2, BRCA1), 3q (PIK3CA), X (AR), and 6q (c-MYB), as well as loss on 9p (CDKN2A). Additional alterations included copy number gains of ARID1A (chr1p). The whole-genome CNV profile confirmed these focal events against a background ploidy of 1.83 and a cfDNA tumor fraction of 0.38, as estimated by iChor analysis. Short-tandem microsatellite (STR) profiling showed propagated PDX tumor matched with the patient’s tumor (greater than 80% match) (Fig. 3). No evidence of contamination or mixed genetic profiles was detected. These results confirm the concordance between ctDNA-based CNV profiling and tissue-based molecular characterization, supporting the presence of key oncogenic drivers in the novel USCIBC3 IBC PDX model.

### Tumor growth characteristics of the PDX and response to alpelisib/everolimus

**Figure 4:**
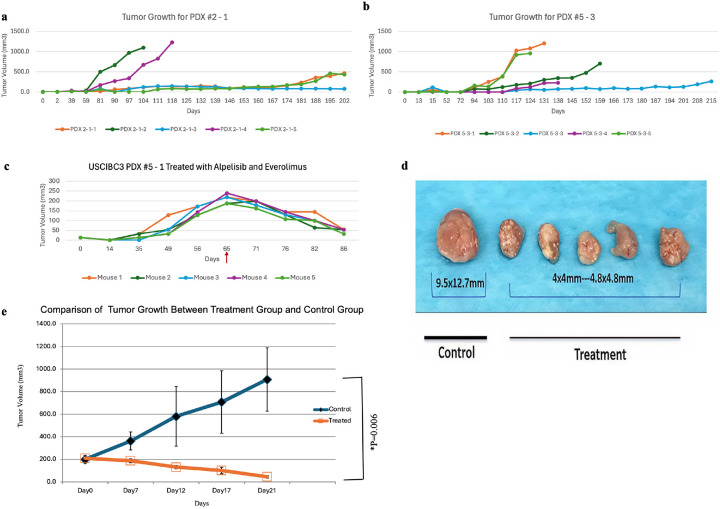
Tumor Growth Inhibition With Alpelisib and Everolimus in the USCIBC3 IBC PDX Model a-b. Tumor growth plotted as tumor volume over time. Tumor engraftment rate was 12/15 (80%) and median tumor volume doubling was 24.5 days (range 9.2–175 days) for n=15 untreated controls. Notation: PDX 2 (first generation)-1(second generation)-1third generation). c. Treatment of mice with alpelisib and everolimus began on day 65 (arrow). Mice were then observed for 21 days after treatment start and tumor volume measured. d. Comparison of tumor growth between treatment and control group. Tumor size of treatment and control groups began at 200mm3, treatment group was terminated at day 21 and control mouse tumor was harvested at the same time. Treatment group received oral 100ul of 20mg/kg alpelisib and 10mg/kg everolimus. e. Average tumor volume in control vs treatment groups over 21 days. Time of tumor growth comparison began with tumor volume at 200mm3. N = 8 in control group. N = 5 in treatment group. Using student's T test, p = 0.006

Third-generation USCIBC3 PDX mice were treated with alpelisib and everolimus (n = 5) or left untreated as controls (n = 8) to evaluate treatment response. The engraftment rate was 12/15 (80%) and the median tumor volume doubling was 24.5 days (range 9.2–175 days) for n = 15 untreated controls (Fig. 4A–C). Due to the PIK3CA mutation (p.H1047R) 3rd generation mice were treated with alpelisib and everolimus to block signaling of the PI3K/AKT/mTOR pathway. Figure 4D shows that all treated tumors decreased in size compared to an example of an untreated tumor. Figure 4E shows that n = 5 PDX treated with albelisib and everolimus were significantly smaller than n = 8 untreated PDX (p = 0.006). No definite metastases were found on necropsy in any mice as the model tended to produce tumors meeting predefined criteria for euthanasia in most mice between 100–150 days.

### Immunohistochemistry of treated and untreated PDX

**Figure 5:**
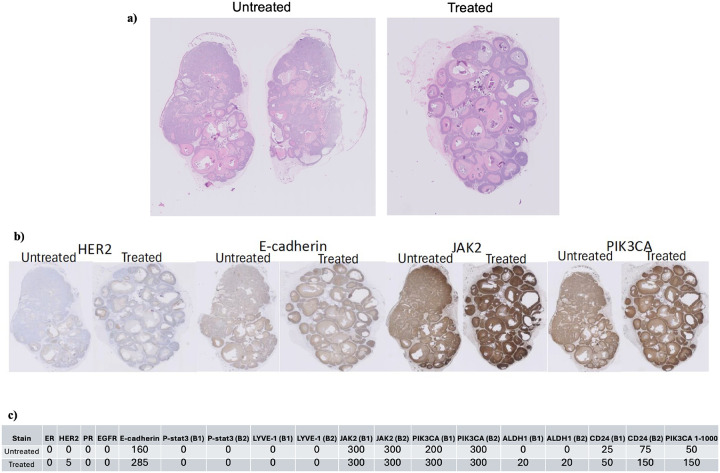
Histopathologic and Immunohistochemical Characterization of USCIBC3 PDX Before and After Treatment with Alpelisib and Everolimus (a-b) Representative hematoxylin and eosin (H&E) and immunohistochemical (IHC) staining of USCIBC3 PDX tumor sections. Sample images of IHC slides comparing protein expression in untreated vs. treated tissue. After treatment, tumor expressed higher levels of HER2, E-cadherin, and PIK3CA c. H-score analysis demonstrated strong membranous E-cadherin expression in both untreated and treated tumors, confirming preservation of epithelial identity. HER2 expression increased modestly following treatment, while ER, PR, and EGFR remained negative. Activation markers JAK2 and PIK3CA were highly expressed in both conditions, consistent with pathway engagement.

Figure 5 shows sample images of IHC slides comparing protein expression in untreated vs treated tissue. The associated table provides H-scores showing differences in protein expression between the untreated (control) vs. treatment groups. Post-treatment, PDX tumor expressed higher levels of HER2, E-cadherin, and PIK3CA compared to untreated tissue. JAK2 was strongly expressed in both treated and untreated tumors. TCHP resistant PDX tumor cells downregulated HER2 expression, which was re-expressed after treatment with alpelisib and everolimus.

#### 10X Genomics Single Cell Sequencing

**Figure 6:**
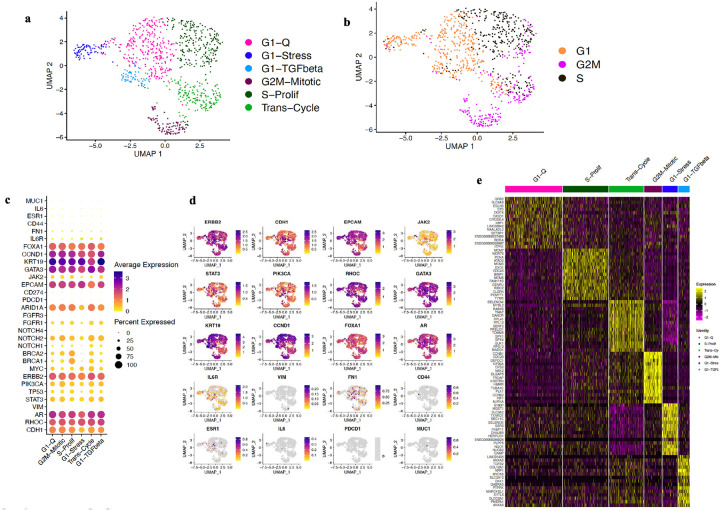
Cellular Heterogeneity and Transcriptional Programs Identified by Single-cell RNA Sequencing of the USCIBC3 Inflammatory Breast Cancer PDX a. UMAP visualization showing six transcriptionally distinct clusters (0–5) of tumor cells identified in the USCIBC3 patient-derived xenograft (PDX) by Seurat-based clustering analysis. b. Cell cycle phase distribution of PDX tumor cells, highlighting transcriptional heterogeneity across G1, S, and G2/M phases, indicative of actively cycling tumor populations. c. Dot plot displaying the average expression (color intensity) and percentage of cells expressing key epithelial, luminal, and signaling pathway genes (dot size) across clusters. d. UMAP visualization of selected marker genes illustrating cluster-specific expression patterns associated with epithelial differentiation and oncogenic signaling programs. e. Heatmap of the top differentially expressed genes across clusters demonstrating transcriptional heterogeneity within the tumor.

Single-cell RNA sequencing (scRNA-seq) was performed on dissociated cells from the USCIBC3 PDX tumor to evaluate the transcriptional landscape and cellular heterogeneity of this newly developed HER2positive inflammatory breast cancer model. After quality control, approximately 1,000 high-quality single-cell transcriptomes were retained for downstream analysis. UMAP visualization revealed transcriptionally distinct tumor epithelial clusters with uniform expression of canonical epithelial markers *EPCAM, CDH1*, and *KRT19*, confirming the epithelial origin of the PDX (Fig. 6A;D). The six transcriptionally distinct clusters were subsequently annotated based on cell-cycle enrichment. The major proliferative cluster (S-Phase Proliferative Cells) showed strong expression of DNA replication genes, while the Mitotic Tumor Cells were enriched for G2/M markers. In contrast, G1-Quiescent Tumor Cells (clusters 0, 4, 5) exhibited reduced proliferation signatures, with Secretory-Stress G1 Cells and TGFβ-Signaling/ECM-Interactive Cells displaying distinct functional programs. The Transitional Cycling Cells (cluster 2) contained a mixture of cell cycle states (Fig. 6B).

The PDX tumor cells demonstrated high expression of *ERBB2 (HER2), PIK3CA, CCND1, FOXA1*, and *GATA3*, consistent with luminal epithelial differentiation and hyper-proliferation. Expression of androgen receptor (*AR)* was moderate, while *VIM, MUC1, FN1*, and *CD44* expression were limited, indicating a predominantly epithelial phenotype with minimal epithelial–mesenchymal transition (EMT). Pathway-related transcripts such as *JAK2, STAT3*, and *IL6R* were focally expressed, suggesting engagement of the IL6/JAK/STAT signaling axis implicated in IBC aggressiveness. In contrast, *PDCD1* and *IL6* expression were sparse.

**Figure 7:**
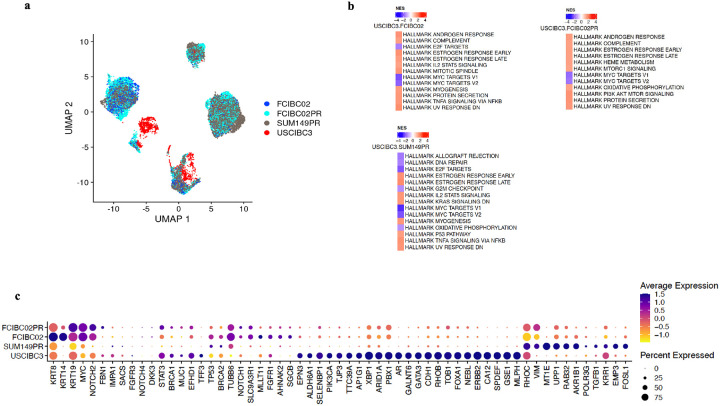
Single-Cell Transcriptomic Comparison of the USCIBC3 PDX With Established IBC Models. a. UMAP visualization comparing single-cell RNA-seq profiles of the USCIBC3 PDX with published datasets for SUM149PR, FC-IBC-02, and FC-IBC-02PR (GSE163397). b. Gene set enrichment analysis comparing USCIBC3 compared to each IBC cell line c. Dot plot of significantly differentially expressed genes between USCIBC3 and each cell line. Dot size reflects the proportion of cells expressing the gene, and color intensity reflects average expression level. Differentially expressed biomarkers grouped into functional categories, including luminal epithelial markers, basal/mesenchymal programs, PI3K/AKT/mTOR pathway components, vesicle-trafficking and ER-stress genes, Notch markers, and ECM remodeling genes underscore the distinct molecular phenotype of USCIBC3 relative to established IBC cell lines.

To assess molecular fidelity relative to established IBC models, we compared the single-cell expression profiles of the USCIBC3 PDX to previously published single-cell RNA sequencing data from the triple-negative IBC cell lines SUM149PR, FC-IBC-02, and FC-IBC-02PR using metareference-based annotation (GEO accession number: GSE163397).^10^ UMAP analysis revealed a distinct transcriptional profile for the USCIBC3 PDX, showing partial similarity to the paclitaxel-resistant triple-negative IBC lines SUM149PR and FC-IBC-02PR (Fig. 7A). Functional markers assessed included luminal differentiation, basal/mesenchymal programs, PI3K/AKT/mTOR signaling, and extracellular matrix remodeling. No gene was universally expressed across all samples. Shared expression of luminal differentiation markers (*KRT8*, *KRT19*, *FOXA1, MUC1*) and *CD44*, reflects retained luminal differentiation programs that have been associated with tumor aggressiveness, metastatic potential, and therapy resistance. The cell lines exhibited higher expression of basal and mesenchymal markers such as *VIM*, *KRT14*, KRT5, *TGFβ1*, *AKR1B1*, and *EMP3*. RhoC expression was highest in paclitaxel-resistant IBC cell lines (SUM149PR and FC-IBC02PR), markedly lower in the parental FC-IBC02, and nearly absent in the USCIBC3 PDX.

This analysis revealed high expression of several genes associated with vesicle trafficking and endoplasmic reticulum stress/secretory pathways (*XBP1, EPN3, AP1G1, SLC9A3R1, MLPH, and RHOB*) supporting a model of enhanced secretory and cell trafficking activity in USCIBC3. GSEA revealed upregulation of PI3K/AKT/mTOR and estrogen response pathways, while MYC signaling was significantly downregulated in the PDX compared with all cell lines (Fig. 7B and Supplementary Fig. 1). To delineate intercellular communication patterns within the untreated tumor microenvironment, we applied CellChat analysis to the scRNA-seq dataset (Supplementary Fig. 2). This revealed intercellular communication characterized by extracellular matrix remodeling signaling networks, including laminin and collagen. TGFβ, JAG1/NOTCH, and CD44-mediated signals were also prominent.

Importantly, the PDX retained robust ERBB2 expression, suggesting a HER2-enriched molecular phenotype. Collectively, these data demonstrate that the USCIBC3 PDX faithfully recapitulates key oncogenic, epithelial, and inflammatory signaling features of the patient’s primary tumor and provides a robust preclinical platform to investigate therapeutic resistance in HER2-positive IBC.

## Discussion

We established a PDX of a HER2 positive IBC tumor with a PIK3CA hotspot mutation (H1047R) refractory to TCHP. TCHP resistant tumor cells downregulated HER2 expression, which was re-expressed after treatment with alpelisib and everolimus. Post-treatment re-expression of HER2 and PIK3CA may represent potential mechanisms of adaptive resistance. Alpelisib and everolimus significantly decreased tumor size in our model. Targeting the PI3K/mTOR pathway may be useful to overcome resistance in HER2 positive IBC with a H1047R mutation in PIK3CA.

Exome sequencing of the inflammatory breast cancer cell line SUM190 (also hormone receptor negative, HER2 amplified) found that it also has a H1047R mutation in PIK3CA and was also noted to be quite sensitive to alpelisib *in vitro*^25^. Jank et al reported that among a cohort of 1691 HER2 positive breast cancer patients who participated in 4 neoadjuvant trials, 17.9% of the cohort had a PIK3CA mutation^26^. The presence of a H1047R mutation is associated with a reduced likelihood of pathologic complete response to neoadjuvant chemotherapy in HER2 positive breast cancer^26^. Garay et al showed that mutations in the kinase domain (H1047R) but not the helical domain increase resistance to HER2 targeted therapy, driven by sustained AKT signaling^27^. Allouchery et al reported that 25% of IBC patients have a PIK3CA mutation identified on biopsy, of which 55% had detectable circulating PIK3CA mutations in their plasma tumor DNA^28^.

Alpelisib is approved for use in hormone receptor positive metastatic breast cancers with a PIK3CA mutation based on the SOLAR-1 trial^29^. Everolimus inhibits the PIK3CA/AKT/mTORC1 pathway downstream of PIK3CA and was approved for use in hormone receptor positive, HER2 negative metastatic breast cancer based on the BOLERO-2 trial^30^. In the BOLERO-1 trial, everolimus plus trastuzumab did not significantly improve progression-free survival over trastuzumab alone in HER2 positive advanced breast cancer^31^. Alpelisib and everolimus are therefore options to address endocrine resistance in metastatic hormone receptor positive, HER2 negative breast cancer. Toxicity is a concern for alpelisib, which has limited its use in the clinic^32^. Zhang et al described a novel PI3K-p110alpha proteolysis targeting chimera (PROTAC) that restored sensitivity to lapatinib in preclinical models of resistant HER2 positive breast cancer^33^. While our data show that targeting the PIK3CA/AKT/mTORC1 pathway decreases tumor growth, further investigations will be required to determine the optimal means of overcoming resistance to HER2 targeted therapy. Several studies have evaluated the potential for targeting genomic mutations with molecularly targeted therapies in treatment refractory cancers^34,35^. USCIBC3 could be highly useful for testing investigational therapeutics and determining how IBC is so treatment resistant. Although USCIBC retains key tumor features and therapy responses, its lack of spontaneous metastasis like many subcutaneous PDX models.^36,37^

USCIBC-3 expressed e-cadherin, one of the defining molecular characteristics of IBC. No tumor spheroids were observed within lymphovascular spaces in this model, unlike MARY-X^38^. The predominantly epithelial phenotype with limited EMT suggests that mesenchymal transition is not the main driver of invasion in IBC, which retains epithelial markers and forms tumor emboli. The post-treatment increase in E-cadherin expression is notable, as E-cadherin retention may promote tumor emboli and lymphovascular invasion.^39,40^ USCIBC exhibited low expression of MUC1, a gene implicated in tumor embolus formation and passive lymphovascular dissemination, in contrast to other IBC PDX models such as MARY-X where it is typically overexpressed.^41^ Rather, this PDX recapitulated the patient’s tumor in that it grew to a large size without dermal lymphatic or nodal invasion.

Like SUM190, USCIBC3 is hormone receptor negative and HER2 amplified with a common PIK3CA mutation in the kinase domain (H1047R), however, we sought to make a PDX rather than a cell line as our primary objective. SUM190 culture is typically done in serum free media with hydrocortisone or insulin supplementation, or more complex medium supplemented with epidermal growth factor (EGF) and lysophosphatidic acid (LPA)^42^. Our lab was able to do short-term culture of USCIBC3 in Primary Cancer Culture System kit according to the manufacturer’s instructions, however, this did not yield an immortalized cell line like SUM190, which spontaneously immortalized as a homogeneous population. The USCIBC3 PDX contains not a pure cancer population, but also cells from the IBC microenvironment as reflected in the heterogeneity demonstrated in our single cell sequencing analysis. This heterogeneity was reflected in six transcriptionally distinct epithelial clusters characterized by variable proliferative activity, metabolic and secretory stress responses, and TGFβ/ECM pathway upregulation, consistent with aggressive, therapy-resistant phenotypes.^43,44^ This diversity underscores dynamic functional states within the tumor. Further optimization will be required for continuous culture and demonstration of stability of subcultures.

Unlike SUM190, the mutation in H1047R was located in exon 21 for USCIBC3, whereas it was in exon 20 for SUM190. The H1047R mutation, located in the kinase domain of the PIK3CA protein, is one of the most common mutant alleles of this gene and has been identified in multiple cancer types, including breast, head and neck, lung, colorectal, and endometrial cancers. It constitutively activates the PI3K catalytic subunit (p110α) by enhancing interaction with its substrate PIP2 at the plasma membrane. Clinically, H1047R and related PIK3CA alterations have been associated with resistance to targeted and endocrine therapies, including trastuzumab and fulvestrant. In a pooled analysis of five prospective trials of HER2-positive breast cancer (n = 967), PIK3CA mutations were linked to lower pathologic complete response (pCR) rates, particularly in the HR+ subgroup (7.6% vs 24.2% in wild type; p < 0.001).^45,46^ STR profiling confirmed that USCIBC3 was derived from our patient’s source primary tumor.

USCIBC3 strongly expressed JAK2. Stevens et al showed that JAK2/STAT3 is a key regulator of inflammatory breast cancer’s aggressive tumor biology. They postulate that a short period of ruxolitinib (RUX), a JAK1 and JAK2 inhibitor, before chemotherapy may help prevent the emergence of a chemotherapy resistant mesenchymal population of cells^10^. However, in a randomized phase II trial, Lynce et al showed that RUX decreased pSTAT3 but no apparent clinical benefit was seen in triple negative IBC patients, likely due to the immune suppressive effects of RUX negating benefit on growth inhibition during treatment alone or in combination with other agents^47^. Our study offers a richer mechanistic framework for adaptive resistance in IBC. USCIBC3 and other PDX models could be useful to dissect the molecular pathways involved with IBC resistance as tumor avatars to help plan future clinical trials based on preclinical data. Humanized mouse PDX models may help overcome some of the limitations of NSG mice by providing the ability to study IBC in the context of a human tumor-immune environment^48^.

We report the establishment of a PDX model from a HER2-positive, TCHP-refractory IBC tumor. The residual disease post-neoadjuvant therapy may be utilized to make useful models to help fight aggressive tumors such as inflammatory breast cancer. While this is the initial report of the characterization of our model, additional mechanistic studies to dissect the effect of JAK2/STAT3 and PIK3CA activation on drug resistance are warranted.

## Supplementary Material

Supplementary Files

This is a list of supplementary files associated with this preprint. Click to download.

- IBCPDXSubmissionSupplementalMaterials.docx

## Synopsis

We have established a novel patient-derived xenograft of a HER2-positive inflammatory breast cancer (IBC) with a H1047R mutation in PIK3CA. Here we report the initial characterization of the model. This model may be employed to better understand the biology of IBC treatment resistance.
